# PI3K/AKT signaling pathway as a critical regulator of epithelial-mesenchymal transition in colorectal tumor cells

**DOI:** 10.1186/s12964-023-01225-x

**Published:** 2023-08-14

**Authors:** Amirhosein Maharati, Meysam Moghbeli

**Affiliations:** 1 Student Research Committee, Faculty of Medicine, Mashhad University of Medical Sciences, Mashhad, Iran; 2 Department of Medical Genetics and Molecular Medicine, School of Medicine, Mashhad University of Medical Sciences, Mashhad, Iran

**Keywords:** Colorectal cancer, PI3K/AKT, EMT, Metastasis, Prognosis

## Abstract

Colorectal cancer (CRC) is one of the most frequent gastrointestinal malignancies that are considered as a global health challenge. Despite many progresses in therapeutic methods, there is still a high rate of mortality rate among CRC patients that is associated with poor prognosis and distant metastasis. Therefore, investigating the molecular mechanisms involved in CRC metastasis can improve the prognosis. Epithelial-mesenchymal transition (EMT) process is considered as one of the main molecular mechanisms involved in CRC metastasis, which can be regulated by various signaling pathways. PI3K/AKT signaling pathway has a key role in CRC cell proliferation and migration. In the present review, we discussed the role of PI3K/AKT pathway CRC metastasis through the regulation of the EMT process. It has been shown that PI3K/AKT pathway can induce the EMT process by down regulation of epithelial markers, while up regulation of mesenchymal markers and EMT-specific transcription factors that promote CRC metastasis. This review can be an effective step toward introducing the PI3K/AKT/EMT axis to predict prognosis as well as a therapeutic target among CRC patients.

Video Abstract

**Supplementary Information:**

The online version contains supplementary material available at 10.1186/s12964-023-01225-x.

## Background

Colorectal cancer (CRC) is the third most common cancer globally and the second cause of cancer-related deaths, with approximately 935,000 deaths in the year 2020 [1]. Since, there is not any significant clinical symptoms in the early stages of CRC, majority of the patients are diagnosed in advanced tumorstages with a poor prognosis [2]. The standard CRC treatment is primarily surgery, while combined adjuvant chemotherapy has increased the overall survival of patients with advanced cancer. Nevertheless, the metastasis and proliferation of tumor cells are the major causes of CRC-related death [3, 4]. Approximately 22% of CRC patients have metastatic CRC with a poor prognosis [5, 6]. Although early-stage CRC patients have a 5-year OS of 80–90%, the survival rates reduce substantially to 40–60% for those with late-stage disease and further decrease to 5–10% among metastatic patients [7]. Therefore, it is required to assess the molecular mechanisms of CRC metastasis to reduce the tumor relapse and distant metastasis among these patients. Epithelial-to-mesenchymal transition (EMT) is a biological process that plays a crucial role in various physiological and pathological conditions such as tissue homeostasis and tumorigenesis. It is characterized by transforming epithelial cells into mesenchymal cells, which decreases their capacity for adhesion and apoptosis while increasing their ability for migration and invasion [8, 9]. EMT process attenuates cell–cell adhesion and downregulates epithelial markers (E-cadherin), whereas it induces cell mobility and the expression of mesenchymal markers (fibronectin, vimentin, and N-cadherin) [10]. It is also implicated in tumor progression, metastasis, and drug resistance. EMT enables tumor cells at the primary tumor site to acquire migratory and invasive capabilities, facilitating their dissemination to distant organs and eventually metastasis [11, 12]. Upregulation of EMT stimulator can induce this cellular process that results in CRC metastasis. These stimulators down regulate the CDH1 while up regulate the mesenchymal factors such as CDH2 and VIM via regulation of EMT-mediated signaling pathways and EMT transcription factors. MicroRNAs (miRNAs) also target mRNAs of EMT-transcription factors. Therefore, down regulation of these miRNAs can be involved in CRC metastasis [13]. A network of signaling pathways, including WNT, NOTCH, and PI3K/AKT modulate the the molecular mechanisms involved in EMT process. The WNT signaling pathway induces EMT by modulating the expression of EMT-associated transcription factors, such as Snail, Slug, and Twist [14, 15]. NOTCH signaling affects EMT by promoting the expression of mesenchymal markers (Vimentin and CDH2) and suppressing epithelial markers (CDH1 and β-catenin) [16]. PI3K/AKT pathway has also a vital role in EMT by activating downstream effectors that regulate cellular processes, including cell survival, migration, and invasion [17, 18]. PI3K/AKT pathway is a crucial signaling cascade involved in regulation of cell growth, survival, and metabolism [19]. It can be activated via the binding of growth factors to receptor tyrosine kinases (RTKs) [20, 21]. RTKs activation recruits and activates the phosphatidylinositol 3-kinase (PI3K) to generate PIP3 that activates the AKT kinase [22]. Additionally, the activated AKT kinase phosphorylates and regulates a wide range of downstream targets such as transcription factors (FOXOs), cell cycle regulators (p21 and p27), and components of the mTOR pathway (mTOR and p70S6K) [23, 24]. AKT can induce EMT by upregulating transcription factors such as Snail, Slug, and Twist, or directly through repressing expression of epithelial markers and promoting mesenchymal markers [25, 26]. For example, PI3K/AKT-induced WDR5 overexpression provoked CRC metastasis via modifying EMT-related markers and enhancing ZNF407 transcription [27]. The CCL20/CXCL8 axis also triggered the PI3K/AKT pathway to promote EMT [28]. Understanding the molecular mechanisms of EMT and its crosstalk with signaling pathways paves the way for the development of targeted therapies to prevent metastasis and improve prognosis among CRC patients. Therefore, in the present review we discussed the role of PI3K/AKT in regulation of EMT process during CRC progression and metastasis (Table 1).

**Table 1:** Role of PI3K/AKT in regulation of EMT process during CRC progression

Study	Year	Axis	Effect on the EMT	Samples	Clinical application
Wei [17]	2019	FAT4/PI3K/AKT/GSK-3B	Induced EMT	100 T 100N^a^ HCT116, LOVO and SW480 cell lines Xenograft model	Therapeutic
Tan [27]	2017	PI3K/AKT/WDR5	Induced EMT	161 T 161N HT-29, SW620, HCT-15, HCT116, and COLO205 cell lines Xenograft model	Therapeutic and prognostic
Pan [29]	2020	miR-328–3p/ Girdin/AKT	Suppressed EMT	HCT116 and SW620 cell lines Xenograft model	Therapeutic
Gao [30]	2022	MOR/AKT	Induced EMT	180 T 180N HCT116, Caco-2, SW480, and LoVo cell lines	Therapeutic and prognostic
Liu [31]	2022	CENPO/AKT	Induced EMT	100 T 100N HCT116 and RKO cell lines Xenograft model	Therapeutic and prognostic
Yang [32]	2018	GLI1/PI3K/AKT/NF-KB	Induced EMT	109 T 35N HT29 and HCT116 cell lines	Therapeutic
Zhang [33]	2021	PI3K/AKT/AGR2	Induced EMT	LoVo, SW480, HT-29, DLD-1, SW48 and HCT116 cell lines Xenograft model	Therapeutic
Li [34]	2020	UCHL3/AKT/SOX12	Induced EMT	8 T 8N Lim1215, DLD1, SW48, HCT116, SW620, and SW480 cell lines Xenograft model	Therapeutic and prognostic
Chen [35]	2021	MYSM1/PI3K/AKT	Suppressed EMT	41 T 41N SW620 and LOVO cell lines Xenograft model	Therapeutic and prognostic
Golhati [36]	2011	mTORC/Rac1/RhoA	Suppressed EMT	18 T 18 M 18N HCT116, SW480 and KM20 cell lines Xenograft model	Therapeutic
Cui [37]	2019	TTN-AS1/ miR-497	Induced EMT	95 T 95N SW480, SW620, HT29, HCT116 cell lines Xenograft model	Therapeutic and prognostic
Liao [38]	2022	KIFC3/PI3K/AKT/mTOR	Induced EMT	HT29, HCT116, SW480, DLD-1 cell lines Xenograft model	Therapeutic
Duan [39]	2018	IMPDH2/AKT/mTOR/FOXO1	Induced EMT	248 T 248N HCT116, SW620, M5, SW480, HT29, DLD-1 and LoVo cell lines Xenograft model	Therapeutic and prognostic
Xu [40]	2020	LACTB/AKT/mTOR	Induced EMT	80 T 80N LOVO, SW480 and HCT116 cell lines Xenograft model	Therapeutic and prognostic
Li [41]	2022	GREM1/PI3K/AKT/mTOR	Induced EMT	55 T 55N SW480 and HCT116 cell lines Xenograft model	Therapeutic and prognostic
Long [42]	2022	ECM1/PI3K/AKT/GSK3B/SNAIL	Induced EMT	75 T 70N NCM460, HCT116, HCT15, HT29, SW480, 293 T, and SW620 cell lines Xenograft model	Therapeutic and prognostic
Zhang [43]	2021	P2X7R/AKT/GSK3B	Induced EMT	SW620 and HCT116 cell lines Xenograft model	Therapeutic
Zhao [44]	2019	CAPS1/PI3K/AKT/GSK3B	Induced EMT	126 T 126N HT29, SW480, DLD1, and SW620 cell lines	Therapeutic and prognostic
Yu [45]	2021	CDX2PTEN/PI3K	Suppressed EMT	161 T 161N RKO, Caco-2, HT-29, SW480 and Lovo cell lines Xenograft model	Therapeutic and prognostic
Shen [46]	2017	CXCL8/PI3K/AKT/NF-KB	Induced EMT	LoVo cell line Xenograft model	Therapeutic
Cheng [47]	2014	CXCL8/ PI3K/AKT	Induced EMT	213 T 213N SW480 and Caco-2 cell lines	Therapeutic
Gao [48]	2019	CCR7/PI3K/AKT	Induced EMT	190 T 190N HCT116, Caco-2, DLD-1, SW620, SW480, HT-29 and LoVo cell lines	Therapeutic and prognostic
Wang [49]	2020	miR425-5p, miR-130b-3p, and miR-25-3p/ PTEN	Induced EMT	17 T 12N HCT116 and SW620 cell lines Xenograft model	Therapeutic
Wei [50]	2019	CCL22/PI3K/AKT	Induced EMT	68 T 68N DLD1 and HT29 cell lines	Therapeutic and prognostic
Chen [51]	2021	Id4/PI3K/AKT	Induced EMT	HCT116 cell line Xenograft model	Therapeutic
Yu [52]	2019	GATA1/AKT	Suppressed EMT	74 T 74N HCT-116 and HCT-8 cell lines Xenograft model	Therapeutic and prognostic
Zhang [53]	2020	miR-758/ PAX6	Suppressed EMT	84 T 84N HCT-116 and SW620 cell lines	Therapeutic and prognostic
Cong [54]	2019	miR-760/ FOXA1	Suppressed EMT	54 T 54N SW620, HT29, DLD1 and HCT116 cell lines	Therapeutic and prognostic
Miller [55]	2020	LSD1/AKT	Induced EMT	HT29, SW480, HCT116, LoVo and RKO cell lines	Therapeutic
Zhao [56]	2016	SPOCK1/PI3K/AKT	Suppressed EMT	HCT116, HT29, SW480, and Lovo cell lines Xenograft model	Therapeutic
Chen [57]	2016	STC2/PI3K/AKT	Induced EMT	77 T 77N HT29 cell line Xenograft model	Therapeutic and prognostic
Chen [58]	2019	CLCA4/PI3K/AKT	Induced EMT	64 T 64N SW620 and LoVo cell lines	Therapeutic and prognostic

^a^Tumor (T) tissues, Normal (N) margins

## PI3K/AKT axis

**Fig. 1:**
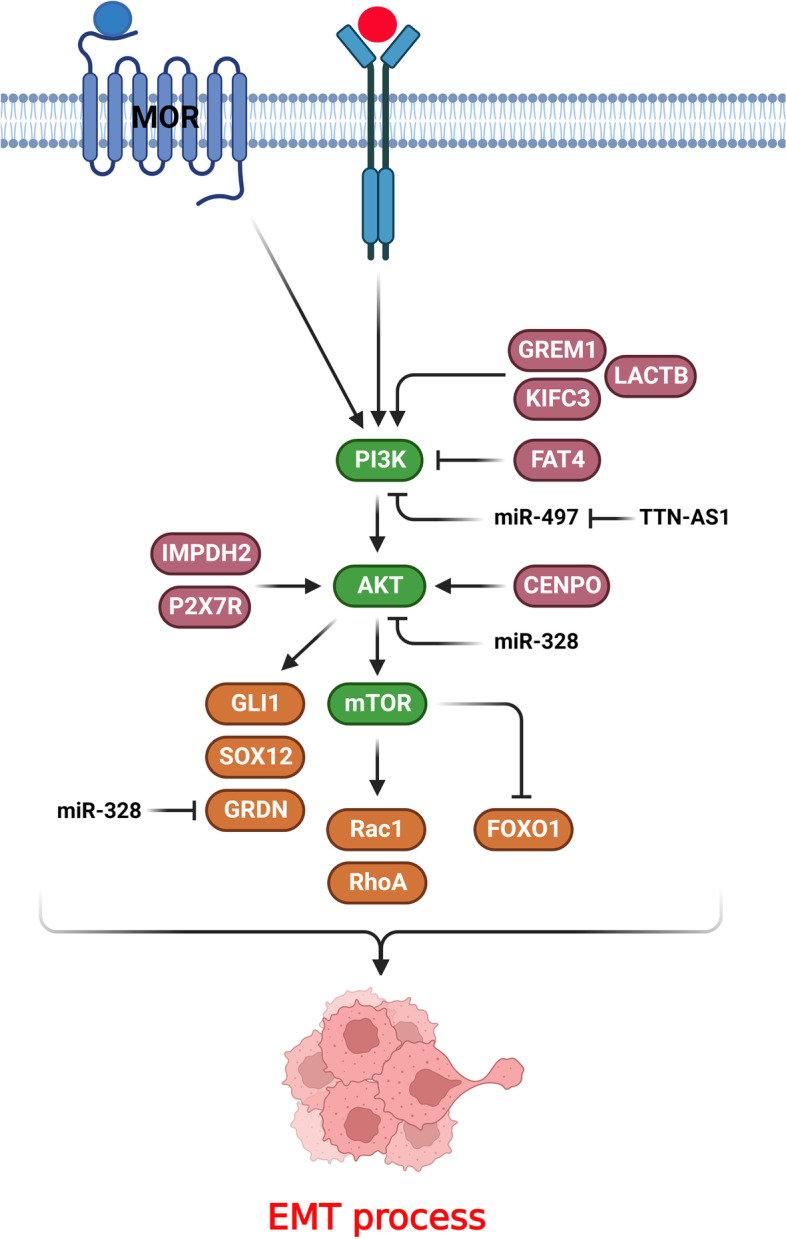
PI3K/AKT/mTOR axis has a pivotal role in regulation of EMT process during CRC progression. (Created with BioRender.com)

PI3K/AKT axis has a pivotal role in regulation of EMT process during CRC progression (Fig. 1). Girdin is an actin-binding protein that is activated by AKT to regulate cytoskeletal remodeling and cell motility. It also promotes AKT signaling by RTKs and G protein-coupled receptors. The p85α subunit of PI3K and phosphorylated Girdin produce the Girdin-p85α complex that stimulates the PI3K/AKT signaling pathway [59]. AKT-dependent Girdin phosphorylation provokes the biological role of Girdin [60]. MiR-328–3p suppressed the EMT in CRC cells through CDH1 up regulation and negative regulation of Snail, Vimentin, and CDH2. Additionally, miR-328–3p repressed the PI3K/AKT pathway by reducing p-AKT and p-Girdin in metastatic liver specimens. Therefore, miR-328–3p stimulated the PI3K/AKT signaling axis via regulating Girdin [29]. Mu-opioid receptor (MOR) belongs to the G protein-coupled receptor family that binds to opioids, including heroin, fentanyl, and morphine [61]. MOR is extensively expressed in various normal tissues as well as human cancers, and its upregulation has been associated with a poor prognosis in cancer patients [62–64]. MOR depletion dramatically inhibited EMT and migration of CRC cells and downregulated p-AKT, which can be restored by SC79 as an activator of PI3K/AKT [30]. Collagen as a key extracellular matrix protein facilitates tumor CRC progression by stimulating the PI3K/AKT signaling axis through the integrin α2β1. PI3K/AKT also promoted EMT by snail up regulation and CDH1 down regulation that resulted in CRC metastasis [65]. Matrix metalloproteinases (MMPs) belong to the zinc-containing endopeptidases family that is implicated in cancer progression via remodelling of the extracellular matrix [66–68]. There was MMP1 up regulation in CRC samples that was correlated with poor prognosis. MMP1 down regulation inhibited AKT pathway and affected the levels of CDH1, CDH2, vimentin, and Twist1 expressions [69]. CENPO modulates the recovery of spindle injury and cell apoptosis by preventing premature separation of chromatids [70]. The centromere has a pivotal role in chromosome separation during cell prolifaration [71]. Dysregulation of the centromere protein CENPO results in chromosomal aneuploidy and abormal cell division [72]. A significant upregulation of CENPO was detected in CRC patients that were associated with decreased survival time. CENPO inhibition also reduced colorectal tumor cell growth and migration. Loss of the CENPO up regulated E-cadherin while down regulated Vimentin and N-cadherin. The suppression of CENPO negatively regulated AKT phosphorylation, CCND1, PIK3CA, and enhanced MAPK9 expression [31]. HH/GLI signaling pathway is a critical modulator of cancer progression as it is involved in cancer stem cell differentiation, metastasis, survival, and growth [73–77]. There was upregulation of GLI1 that was associated with an invasive phenotype and poor prognosis in CRC patients. GLI1 targeted the PI3K/AKT/NFκB pathways to regulate the metastatic features of CRC cells, which reduced the survival rate of CRC patients. The loss of GLI1 also led to CDH1 upregulation while Snail and vimentin down regulations in CRC cells. Additionally, AKT inhibition significantly repressed GLI1. Therefore, PI3K/AKT axis was an upstream effector of GLI1 and induced CRC characteristics by activating GLI1. PI3K/AKT/NF-κB signaling pathway ameliorated the growth and metastasis and attenuated the survival time of CRC cells via GLI1 up regulation [32].

Anterior gradient-2 (AGR2) belongs to disulfide isomerase protein family that has a pivotal role in cancer progression. It encompasses a KTEL motif that is a carboxy-terminal endoplasmic reticulum (ER) retention pattern [78–82]. KTEL facilitates the AGR2 attachment to KDEL receptors on the Golgi for retrograde transport and localization in the ER, where it promotes appropriate protein folding [83, 84]. It has been reported that intracellular AGR2 increased CRC metastasis via inducing EMT, resulting in SLUG and SNAIL up regulations. There was AGR2 upregulation following PGE2 stimulus through the EP4-PI3K-AKT axis, indicating its critical role in the regulation of PGE2-mediated EMT and the crosstalk between TAMs and tumor cells [33]. Ubiquitination is a critical mechanism that regulates protein functions posttranslationally [85, 86]. Deubiquitinating enzymes (DUBs), including UCHL3 are pivotal modulators of the ubiquitination [87–89]. There was UCHL3 up regulation in CRC tissues that was correlated with poor prognosis. UCHL3 induced the growth, invasion, and malignancy of CRC cells. Moreover, suppression of UCHL3 led to E-cadherin upregulation, whereas vimentin, CDH2, Slug, Snail, and ZEB1 down regulations. Therefore, UCHL3 triggerd the proliferation of CRC cells via AKT-induced SOX12 over expression [34]. MYSM1 excludes monoubiquitin from H2AK119ub1 and activates transcription in cooperation with histone acetylation [90]. Dysregulation of MYSM1 results in immune system disorders, anemia, and infammatory reactions, as well as various tissue dysfunctions [91–95]. MYSM1 inhibited the CRC progression via miR-200/CDH1 and inhibition of PI3K/AKT axis [35].

## PI3K/AKT/mTOR axis

The mTOR as one of the main effectors of PI3K/AKT axis has a pivotal role in regulation of EMT process during CRC progression (Fig. 1). Mammalian target of rapamycin (mTOR) is one of the main effectors of PI3K/AKT pathway that regulates cell proliferation, apoptosis, and metabolism. Rac1 and RhoA stimulation regulate the primary steps of the tumor metastasis via actin rearrangement and cell migration [96]. Rac1 triggers lamellipodia production, while RhoA forms cell–cell adhesions and actin stress fibers. mTORC1/2 modulates F-actin reorganization and lamellipodia formation in tumor cells [97]. There was an association between Raptor, Rictor, and mTOR expression and higher stages of CRC. There were Raptor, Rictor, and mTOR up regulations in matastatic CRC samples. mTORC1/2 suppression mitigated the invasion and migration of CRCs, probably through regulating the rearrangement of the cytoskeleton and deactivating Rac1 and RhoA. Moreover, hindering mTORC1/2 enhanced E-cadherin, cell–cell adhesion, and oxaliplatin-induced apoptosis while inhibited lamellipodia formation, fibronectin, SMA, vimentin, and MMP-9 [36]. There was significant up regulation of TTN-AS1 in CRC tissues that was associated with lymph node involvement, TNM stage, and poor prognosis. TTN-AS1 promoted CRC cell proliferation and invasion. TTN-AS1 stimulated PI3K/AKT/mTOR axis partly via targeting miR-497 in CRC cells [37]. Kinesins (KIFs) are motor proteins that have a critical role in intracellular transportation of mRNAs, protein complexes, and organelles through ATP molecules along microtubules. They also modulate the spindle and chromosomal dynamics throughout meiosis and mitosis [98, 99]. KIF2A inhibition promotes apoptosis by PI3K/AKT suppression in tumor cells [100]. There was KIFC3 up regulation in CRC tissues and cell lines, and its suppression decreased invasion of CRC cells. KIFC3 induced the expression of MMP2/9 and mesenchymal-related markers. KIFC3 also phosphorylated mTOR, AKT, and PI3K. Moreover, there was a positive association between KIFC3 and aggressiveness of CRC cells through EMT and the PI3K/AKT/mTOR pathway [38]. Inosine 5′-monophosphate dehydrogenase (IMPDH) facilitates the generation of xanthosine monophosphate that is a vital process in the guanine synthesis [101]. IMPDH modulates the guanine nucleotide levels and play an importat role in synthesis of RNA and DNA. IMPDH2 induced the EMT, invasion, and growth in CRC cells via up regulating Ki-67 and cyclin D1 and down regulating p27Kip1 and p21Cip1. IMPDH2 promoted the G1/S transition via activating AKT and mTOR and FOXO1 down regulation [39].

Beta-lactamase-like (LACTB) modulates the membrane organization in mitochondria that affects the lipid metabolism and oxidative phosphorylation. Autophagy is a critical homeostatic process that degrades cellular proteins, organelles, and cytoplasmic constituents to balance the consumption and supply of energy under stress conditions [102]. Inhibition of LACTB mitigated the autophagy and promoted the EMT and invasion in CRC cells. LACTB regulated the growth of tumor cells via 4E-BP1, C-Myc, and CCND1 by the PI3K/AKT/mTOR axis. LACTB promoted epithelial polarity and amplified cell–cell junctions to suppress EMT via autophagy. LACTB provoked autophagy via modulating PIK3R3 expression and repressing EMT and cell growth via the regulation of PI3K/AKT/mTOR axis [40]. FAT4 facilitated autophagy by suppressing PI3K, p-AKT, and mTOR. Inhibition of FAT4 also suppressed autophagy while enhanced invasion and EMT in CRC cells. FAT4 regulated the EMT via targeting Twist1 and E-cadherin. Therefore, FAT4 ameliorated autophagy and attenuated the EMT through the PI3K/AKT/GSK-3β and mTOR pathways [17]. Gremlin-1 (GREM1) is a glycoprotein that is classified as a member of the DAN/Cerberus protein family that includes various proteins such as VEGF and TGF-β [103]. GREM1 stimulates organ fibrosis as a key step within the EMT process [104, 105]. Endoplasmic reticulum (ER) participates in protein folding, protein transport, calcium storage, and lipid biosynthesis [106, 107]. Stress stimuli disturb the ER proteostasis, which is followed by unfolded protein response (UPR) stimulation. Three ER membrane receptors, including ATF6, PERK, and IRE1α rescue ER proteostasis, induce cell death, and initiate UPR signaling [108–112]. GREM1 ameliorated the UPR-induced EMT in CRC cells through ATF6 up regulation while ATF4 down regulation. GREM1 also regulated the ATF6 and ATF4 expressions via the VEGF and BMP pathways. There was GREM1 up regulation in advanced stage CRC patients that was correlated with an unfavorable prognosis. Additionally, GREM1 stimulated the PI3K/AKT/mTOR pathway as a target of the VEGF-VEGFR2 axis, which up regulated the ATF6 [41].

## PI3K/AKT/GSK-3β axis

**Fig. 2:**
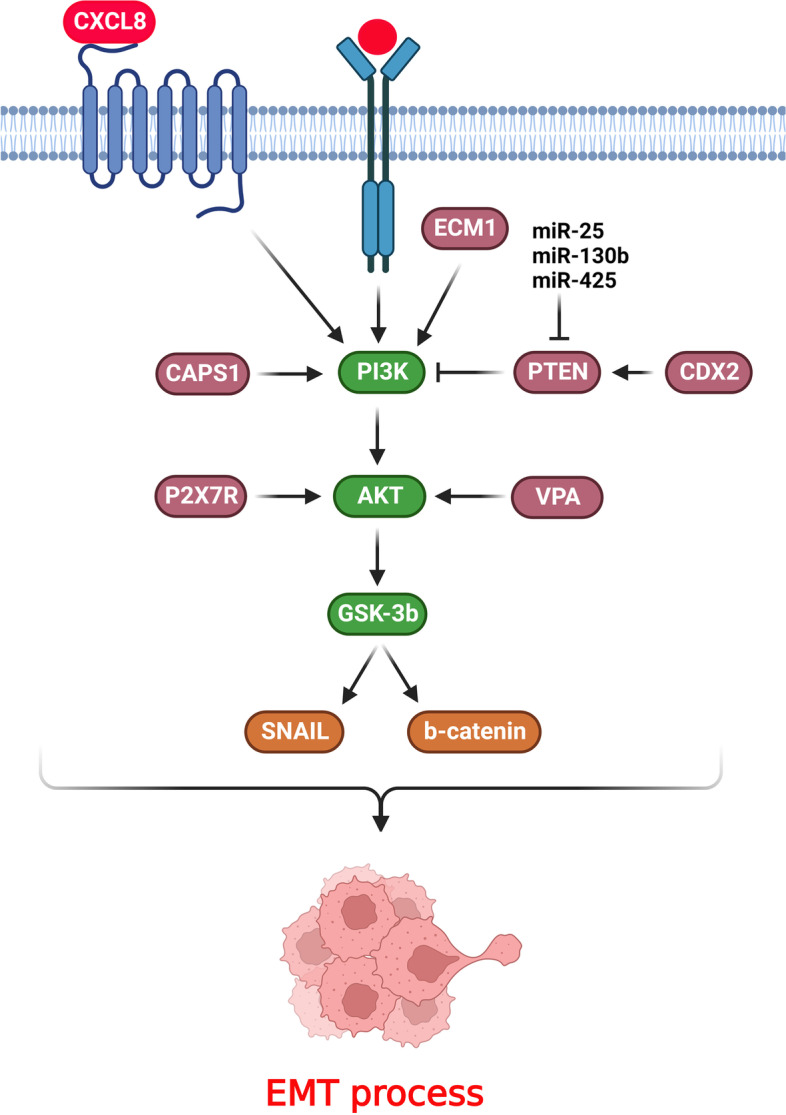
PI3K/AKT/GSK-3β axis has a pivotal role in regulation of EMT process during CRC progression. (Created with BioRender.com)

GSK-3β is a serine-threonine kinase that inhibits glucose homeostasis to regulate ER-stress and apoptotic pathways. GSK-3β as one of the main effectors of PI3K/AKT axis has a pivotal role in regulation of EMT process during CRC progression (Fig. 2). Extracellular matrix protein 1 (ECM1), as a secretory glycoprotein, regulates numerous cellular mechanisms such as angiogenesis, epithelial cell growth, and tumor progression [113]. There was ECM1 up regulation in CRC cancer tissues that was correlated with tumor size, lymph node metastasis, and TNM staging. It also promoted the invasion and migration of CRC tumor cells. ECM1 inhibition down regulated Snail, pGSK3β, and p-AKT in CRC cells. Additionally, ECM1 induced CRC metastasis by promoting EMT via modulating the PI3K/AKT/GSK3β/Snail pathway [42]. P2X purine receptors are ATP-dependent cation channel receptors that regulate potassium ions outflow and sodium and calcium ions influx [114–116]. ATP and its analogs, BzATP, induced the growth and EMT of CRC cells via activating the P2X7R that stimulated the PI3K/AKT/GSK-3β/β-catenin axis. ATP and BzATP triggered the P2X7R to down regulate CDH1, while up regulated the fibronectin, Snail, and Vimentin. P2X7R also phosphorylated GSK-3β and AKT and increased the growth of CRC cells [43]. Snail is a critical suppressor of CDH1 that can be modulated by ERK, TGFβ, and AKT/GSK-3β signaling pathways [117–121]. Exocytosis is a mechanism that delivers various substances between the plasma and intercellular membrane [122]. Calcium-dependent secretion activator 1 (CAPS1) is involved in exocytosis that induced metastasis in CRC cells via PI3K/AKT/GSK3β/Snail axis-induced EMT. CAPS1 interacted with p85 to activate the PI3K/AKT/GSK3β pathway and then increased the expression of Snail, which followed by promoting EMT process and CRC metastasis [44]. Histone deacetylase inhibitors (HDACIs) such as VPA regulate the EMT in numerous cancer cells [123–126]. It has been demonstrated that VPA dramatically increased the EMT in CRC cells. Snail was stabilized by acetylation, and GSK-3β suppression was involved in the VPA-induced EMT of CRC cells. Furtheremore, VPA stabilized and upregulated the Snail via activating the AKT/GSK-3β axis [127]. Caudal-related homoeobox transcription factor 2 (CDX2) is an intestine-related transcription factor involved in the maintenance and growth of intestinal tissue [128]. Loss of CDX2 increased the levels of MMP-9, vimentin, and fibronectin expressions while down regulated the CDH1 and ZO-1. CDX2 knockdown induced EMT-related markers, and PTEN reduced tumor invasion and phosphorylation of AKT and GSK-3β. CDX2 stimulated PTEN expression and subsequent inhibition of the PI3K/AKT/GSK-3β pathway, which led to negative regulation of Snail and β-catenin. β-catenin down regulation reduced the levels of ZEB1, Slug, and Snail expressions while up regulated CDH1 which restrained cell invasion and migration [45].

## Chemokine mediated PI3K/AKT activation

Chemokines are the key regulators of the leukocytes migration that are highly expressed in numerous cancers [129, 130]. They stimulate the PI3K/AKT axis, thereby inhibiting the CDH1/β-catenin complex to facilitate invasion and migration of CRC cells [131–133]. Chemokines are involved in regulation of EMT process via PI3K/AKT axis during CRC progression (Fig. 2). The CCL20 and CXCL8 co-activation restrained invasion, growth, and CDH1 levels while upregulating snail, vimentin, and CDH2. Moreover, the CCL20/CXCL8 axis triggered the PI3K/AKT/ERK pathway to promote EMT [28]. CXCL8 is an autocrine growth factor that induces tumor proliferation, drug resistance, angiogenesis, and aggressiveness [134, 135]. PI3K/AKT pathway also activates the NF-κB signaling through κBα (IκBα) protein phosphorylation [136]. CXCL8 increased the growth and invasion of CRC cells. CXCL8 induced EMT through the PI3K/AKT/NF-κB pathway by PI3K and NF-κB phosphorylations. Moreover, CXCL8 triggered EMT by downregulating E-cadherin and upregulating α-SMA, vimentin, and N-cadherin through PI3K/AKT/NF-κB pathway [46]. CXCL8 and CCL20 promoted EMT in human CRC cells to preserve cell invasion, migration, and proliferation through inducing the PI3K/AKT-ERK1/2 axis. CXCL8 and CCL20 coexpression was associated with liver metastases and a poor prognosis in CRC patients [47]. EGFR is correlated with poor prognosis, and its downstream pathways, including MAPK and PI3K/AKT, induce tumor development [137, 138]. SLC/CCR7 is a pivotal modulator of the EMT in tumor cells [139]. A remarkable correlation has been indicated between CCR7 up regulation, regional lymph node metastasis, and tumor infiltration. SLC activated CCR7, which resulted in PI3K/AKT stimulation in CRC cells. CCR7 induced cetuximab resistance in CRC cells under the regulation of the EMT process [48].

Liver metastasis is a major challenge that accounts for approximately 70% of CRC deaths [140]. Tumor-associated macrophages (TAMs) are the most common type of immune-related cells that infiltrate into the tumor microenvironment to promote metastasis [141]. The classically activated (M1) and alternatively activated (M2) phenotypes are two polarized subtypes of TAMs [142]. M1 macrophages classically activate immune cells that release type I pro-inflammatory cytokines. M2 macrophages enhance tumor progression via immune suppression, angiogenesis, and metastasis [143, 144]. CRC cells delivered miR-425-5p, miR-130b-3p, and miR-25-3p to TAMs via exosomes following the activation of CXCL12/CXCR4 axis. These miRNAs induced the M2 macrophages via targeting PTEN by the PI3K/Akt pathway, leading to increased metastasis and angiogenesis of CRC cells via promoting EMT and releasing VEGF [49]. There was a positive association between CD163 + M2 macrophages presence and CCL22 expression in CRC tissues. M2 macrophages induced 5-FU resistance in CRC cells by releasing CCL22. M2 macrophages also conveyed CCL22 to cancer cells, leading to the promotion of EMT and 5-FU resistance in CRC cells. Furtheremore, 5-FU inhibited the proliferation of CRC cells with PI3K and AKT dephosphorylation. M2 macrophages impaired the inhibitory effect of 5-FU by activating the PI3K/AKT pathway. Therefore, M2 macrophage-secreted CCL22 counteracted the impact of 5-FU on tumor cells by triggering PI3K/AKT [50].

## Transcription factors and chromatin remodelers

**Fig. 3:**
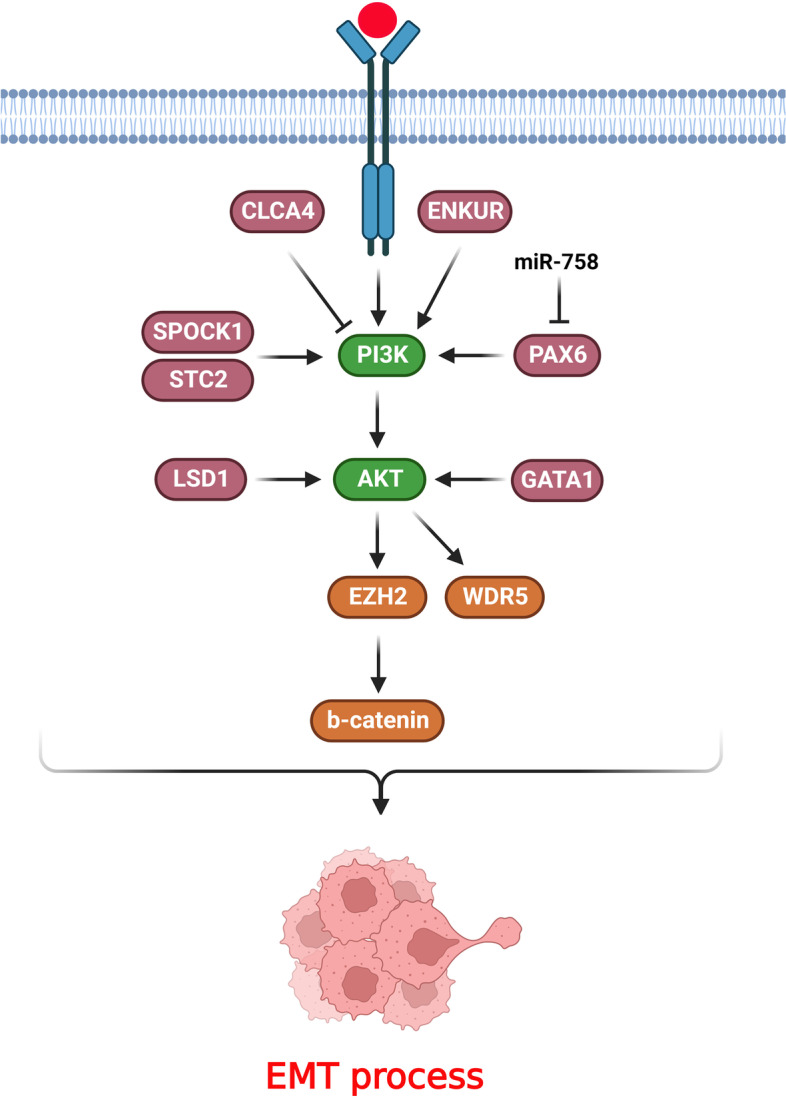
Transcription factors, chromatin remodelers, and ion binding proteins have key roles in EMT process via the regulation of PI3K/AKT pathway during CRC progression. (Created with BioRender.com)

It has been shown that transcription factors and chromatin remodelers have a key role in EMT process via the regulation of PI3K/AKT pathway during CRC progression (Fig. 3). DNA-binding (Id) inhibitors are members of the basic helix-loop-helix (bHLH) transcription factor family that have lost their DNA-binding domain [145]. The Id family critically regulates cell apoptosis, growth, and differentiation [146, 147]. Id4 dramatically suppressed tumor proliferation and metastasis in the xenograft model. The Id4-transfected cells exerted p21 and p27 up regulation, which was followed by cell cycle arrest at the G0/G1. Id4 inactivated AKT and PI3K, suppressing CRC cell proliferation through modulating PI3K/AKT signaling. Id4 mitigated the EMT process as it up regulated TIMP1/2 and down regulated the snail, slug, twist, β-catenin, MMP2, and MMP7 [51]. GATA1 is a critical modulator of erythroid cell apoptosis, proliferation, and differentiation. It has been indicated that GATA1 was up regulated in breast cancer and increased VEGF-induced tumor angiogenesis and proliferation via interaction with the SET7 histone methyltransferase [148, 149]. GATA1 promotes EMT in cancer cells by CDH1 down regulation [150]. There was GATA1 up regulation in CRC tissues, which was associated with poor prognosis. GATA1 provoked CRC cell invasion and growth through AKT phosphorylation [52]. PAX6 as a highly preserved transcription factor is a pivotal regulator of human tumor progression [151]. There was miR-758 downregulation in CRC tissues that was correlated with malignant features and an unfavorable prognosis. MiR-758 suppressed the cell survival and metastasis in CRC cells. It also repressed the EMT and PI3K/AKT axis and promoted apoptosis via Bcl-2 and Bax in CRC. Therefore, miR-758 inhibited cell metastasis and EMT in CRC by targeting PAX6 and inhibition of PI3K/AKT pathway [53]. FOXA1 exhibits oncogenic function in numerous malignancies by modulating cell cycle, growth, and death [152, 153]. There was a significant miR-760 down regulation in CRC, which was correlated with a poor prognosis. MiR-760 targeted the FOXA1 to inhibit the growth, migration, and aggressiveness of CRC cells via modulating EMT and PI3K/AKT [54].

Polycomb repressive complex 2 (PRC2) is involved in tumor progression and tissue homeostasis by modulating the chromatin remodeling. Enhancer of zeste homolog 2 (EZH2) is one of the principal constituents of the PRC2 complex. EZH2 regulates the trimethylation of histone 3 at lysine 27 (H3K27me3), thus repressing transcription. AKT phosphorylated EZH2 following PI3K/AKT axis activation, which is necessary for the cross talk between methylate β-catenin and EZH2. Phosphorylated EZH2 enhanced the β-catenin function, followed by regulating genes involved in metabolic processes and cell migration. Additionally, PI3K/AKT as a critical activated axis in CRC patients led to EZH2 phosphorylation at S21 (pS21-EZH2) [154]. LSD1 belongs to the RE1 silencing transcription factor corepressor (CoREST) complex that includes the RCOR1 as a scaffolding protein and histone deacetylase 1 and 2 (HDAC1/2) as chromatin-modifying subunits [155–157]. CoREST regulates the acetylation of active chromatin to sustain a restrained chromatin phase. LSD1 is implicated in demethylation of H3K4me2 in the promoter of epithelial genes to promote CRC [158–160]. There was significant upregulation of LSD1 in patients who carried PIK3CA mutation in gastrointestinal tumor tissues. LSD1 decreased the proliferation of PIK3CA-mutant colorectal and stomach cancer cells. LSD1 also modulated the phosphorylation of AKT and EMT via CoREST complex. Therefore, LSD1 was up regulated upon PIK3CA mutation that resulted in tumor invasion and EMT features [55]. WDR5 is a highly preserved subunit of COMPASS-related complexes that are involved in H3K4me3 [161, 162]. WDR5 plays an important role in embryonic cell self-renewal and the reprogramming of somatic cells [163, 164]. WDR5 up regulation was prominently associated with an unfavorable prognosis in non-metastatic CRC tissues. Overexpression of WDR5 has also been indicated to trigger CRC metastasis. PI3K/AKT-induced WDR5 expression provoked CRC metastasis via modifying EMT-related markers and up regulating ZNF407 [27].

## Ion binding proteins

Ion binding proteins have a key role in EMT process via the regulation of PI3K/AKT pathway during CRC progression (Fig. 3). SPOCK1 belongs to the Ca2 + -binding proteoglycan family that has an important regulatory role in metastasis, cell cycle, and DNA repair [165–167]. Decreased expression of SPOCK1 markedly mitigated the migration and aggressiveness of CRC cells by impairing the EMT. The loss of SPOCK1 also down regulated the p-Akt and p-PI3K [56]. The stanniocalcin 1 (STC1) and stanniocalcin 2 (STC2) glycoprotein hormones modulate the secretion of phosphate and calcium [168]. STC2 has a vital role in cell–cell interactions between normal colon epithelia and tumor cells [169]. STC2 expression has been correlated with tumor stage and survival time in CRC patients. It also induced the EMT and migration of CRC cells. STC2 accelerated the tumorigenesis and development of tumor cells via the EMT process by stimulating the PI3K/AKT and ERK/MEK pathways. STC2 up regulated p-ERK, p-MEK, p-AKT, PI3K, and Ras upon altering EMT markers [57]. CaM-binding protein is encoded by ENKUR and interplays with the p85 component of PI3K. p85 induces the PI3K signaling cascade to accelerate tumor cell proliferation, metabolism, and survival while inhibited apoptosis [170–172]. ENKUR exhibited a tumor suppressive role in CRC cells as it modulated tumor growth, migration, and invasion. Down regulation of ENKUR activated the PI3K/Akt signaling pathway in CRC cells. Additionally, inhibition of ENKUR downregulated E-cadherin while upregulated vimentin and N-cadherin in CRC cells [173]. Calcium-activated chloride channel (CLCA) modulators are proteins that have a symmetrical multiple cysteine patterns in the terminal tail [174]. Reduced expression of CLCA4 accelerates cell proliferation and metastasis via modulating EMT [175–177]. CLCA4 inhibited the PI3K-AKT signaling and EMT in CRC cells. CLCA4 suppression was also associated with metastasis to lymph nodes in CRC patients. CLCA4 repressed the invasion and migration of CRC cells by attenuating EMT and PI3K/AKT signaling inactivation [58].

## Conclusions

EMT process is considered as one of the main molecular mechanisms involved in tumor metastasis. This process can be directly or indirectly regulated by signaling pathways. It has been reported that the PI3K/AKT pathway plays a key role in promotion of the EMT process during CRC progression by up regulation of mesenchymal markers and EMT specific transcription factors that result in CRC metastasis. Therefore, PI3K/AKT/EMT axis can be used to predict prognosis and as a suitable therapeutic target in metastatic CRC. Since, PI3K/AKT has a key role in promotion of EMT process, it can be expected that the clinical monoclonal antibodies such as Cetuximab and Panitumumab that can target the RTKs as the main triggers of this pathway can be used as the indirect EMT inhibitors to reduce the CRC metastasis and improve prognosis among these patients.

## Data Availability

The datasets used and/or analyzed during the current study are available from the corresponding author on reasonable request.

## Abbreviations

AGR2: Anterior gradient-2
bHLH: Basic helix-loop-helix
LACTB: Beta-lactamase-like
CLCA: Calcium-activated chloride channel
CAPS1: Calcium-dependent secretion activator 1
CDX2: Caudal-related homoeobox transcription factor 2
CRC: Colorectal cancer
DUBs: Deubiquitinating enzymes
ER: Endoplasmic reticulum
EZH2: Enhancer of zeste homolog 2
EMT: Epithelial-mesenchymal transition
GREM1: Gremlin-1
HDAC1/2: Histone deacetylase 1 and 2
HDACIs: Histone deacetylase inhibitors
IMPDH: Inosine 5′-monophosphate dehydrogenase
KIFs: Kinesins
mTOR: Mammalian target of rapamycin
MMPs: Matrix metalloproteinases
MOR: Mu-opioid receptor
PI3K: Phosphatidylinositol 3-kinase
PRC2: Polycomb repressive complex 2
CoREST: RE1 silencing transcription factor corepressor
RTKs: Receptor tyrosine kinases
STC1: Stanniocalcin 1
TAMs: Tumor-associated macrophages
UPR: Unfolded protein response
